# What is an estimand & how does it relate to quantifying the effect of treatment on patient-reported quality of life outcomes in clinical trials?

**DOI:** 10.1186/s41687-020-00218-5

**Published:** 2020-08-24

**Authors:** Rachael Lawrance, Evgeny Degtyarev, Philip Griffiths, Peter Trask, Helen Lau, Denise D’Alessio, Ingolf Griebsch, Gudrun Wallenstein, Kim Cocks, Kaspar Rufibach

**Affiliations:** 1 Adelphi Values Ltd, Bollington, UK; 2 Novartis Pharma AG, Basel, Switzerland; 3 Genentech, San Francisco, CA USA; 4 Novartis Pharmaceuticals Corporation, East Hanover, NJ USA; 5 Boehringer-Ingelheim International GmbH, Ingelheim, Germany; 6 F.Hoffmann-La Roche, Basel, Switzerland

**Keywords:** Estimand, Treatment effect, HRQoL, PRO, ICH, Clinical trial, Design, Objective, Patient

## Abstract

**Background:**

Published in 2019, a new addendum to the ICH E9 guideline presents the estimand framework as a systematic approach to ensure alignment among clinical trial objectives, trial execution/conduct, statistical analyses, and interpretation of results. The use of the estimand framework for describing clinical trial objectives has yet to be extensively considered in the context of patient-reported outcomes (PROs). We discuss the application of the estimand framework to PRO objectives when designing clinical trials in the future, with a focus on PRO outcomes in oncology trial settings as our example.

**Main:**

We describe the components of an estimand and take a naïve PRO trial objective to illustrate how to apply attributes described in the estimand framework to inform construction of a detailed clinical trial objective and its related estimand. We discuss identifying potential post-randomization events that alter the interpretation of the endpoint or render its observation impossible (also defined as intercurrent events) in the context of PRO endpoints, and the implications of how to handle intercurrent events in the construction of the PRO objective. Using a simple objective statement, ***“What is the effect of treatment X on patient’s quality of life?”,*** we build up an example estimand statement and also use a previously published phase III oncology clinical trial to illustrate how an estimand for a PRO objective could have been written to align to the estimate framework.

**Conclusion:**

The use of the estimand framework, as described in the new ICH E9 (R1) addendum guideline will become a key common framework for developing clinical trial objectives for evaluating effects of treatment. In the context of considering PROs, the framework provides an opportunity to more precisely specify and build the rationale for patient-focused objectives. This will help to ensure that clinical trials used for registration are designed and analysed appropriately, enabling all stakeholders to accurately interpret conclusions about the treatment effects for patient-focused outcomes.

## Background

Patient input and incorporating the patient’s perspective within clinical trials is increasingly being recognized as a critical component of achieving patient-centricity in quality health care [1]. As such, assessment of patient-reported-outcomes (PROs), including health-related quality of life (HRQoL), have an important role in clinical trials across disease areas as a way to quantify the effects of treatment exposure on how a patient feels or functions.

The International Committee for Harmonization of Technical Requirements for Pharmaceuticals for Human Use (ICH) is a global organisation that brings together regulatory authorities and pharmaceutical industry in a mission to ensure development and registration of high-quality medicines. ICH issues and maintains numerous guidance documents used internationally in the design and conduct of clinical trials. A key guideline relating to design and analysis of clinical trials is ICH E9 “Statistical Principals for Clinical Studies”. ICH has recognised that a precise definition of the scientific question of interest is required to ensure alignment between objectives, trial design, data collection, analysis and interpretation and that this alignment has not always been observed in the past. In November 2019 the ICH issued the final version of an addendum (R1) to ICH E9, ICH E9(R1) [2], introducing the estimand framework to precisely describe the treatment effect of interest in a randomised clinical trial. An estimand is defined in the guidance as *“...the target of estimation to address the scientific question of interest posed by the trial objective”* and has five attributes – population, treatment, variable (endpoint), intercurrent events (defined as events that can occur post-randomization and preclude or affect the interpretation of the variable) and the summary measure. An example of an intercurrent event in a clinical trial context could be discontinuation of treatment.

While the estimand describes the target of estimation, in practice, teams designing clinical trials are unlikely to come up with a clear and complete objective without discussing the estimand attributes and, in particular, potential intercurrent events. Hence, defining study objectives should be an iterative process, being fine-tuned using the estimand framework as a tool to facilitate such discussion. The clear trial objective would then directly imply how key intercurrent events will be handled and what is the treatment effect of interest. The goal of clarifying the clinical research question more precisely is not new, however, and other concepts such as the PICO model have been previously proposed [3]. The estimand framework differs from the PICO model as it highlights the importance of intercurrent events ensuring that summary measures used to compare treatment conditions are made explicit. It may also in the future potential lead to increased standardisation. The lack of standardized analysis and interpretation of PRO data has been recognized by a number of focus groups working with PRO data in various areas. Within oncology, for example the lack of standardisation is being addressed by a multi-stakeholder, international consortium, SISAQOL [4]. The estimand framework provides common language which can be applied to the initial SISAQOL recommendations, which have focused on developing a taxonomy of research objectives that can be matched with appropriate statistical [5] Extending these to describe more fully within the estimand framework will provide a clear direction for implementation of these recommendations.

The purpose of this article is to introduce the reader to the ICH E9 (R1) addendum guideline, through providing both an explanation, and relevant examples, for the application of the estimand framework as it relates to patient-reported-outcomes included in randomized clinical trials.

## The estimand framework; an introduction & defining intercurrent events

The estimand framework highlights that different treatment effects may be of interest and clarity is required to define *what* the research wants to estimate (estimand). A precise clinical trial objective is required to define an estimand. However, in practice, the scientific research question or questions relating to patient quality of life outcomes may not start as precise objectives. In an iterative process, using the attributes of an estimand can allow a precise clinical trial objective to be developed, which, in turn, can lead to a well-defined and consistent estimand.

**Fig. 1:**
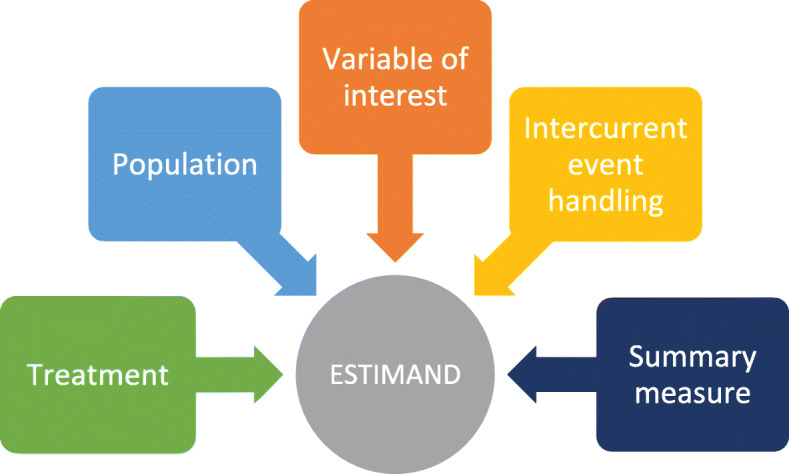
The five attributes of an estimand according to the ICH E9 (R1) addendum

The estimand framework defines five attributes of an estimand (illustrated in Fig. 1), and we will show how these attributes help to clarify the scientific question of interest based on treatment, population, variable of interest (endpoint), intercurrent event handling and the summary measure. All the attributes of the estimand are inter-related, and thus all aspects must be considered.

**Fig. 2:**
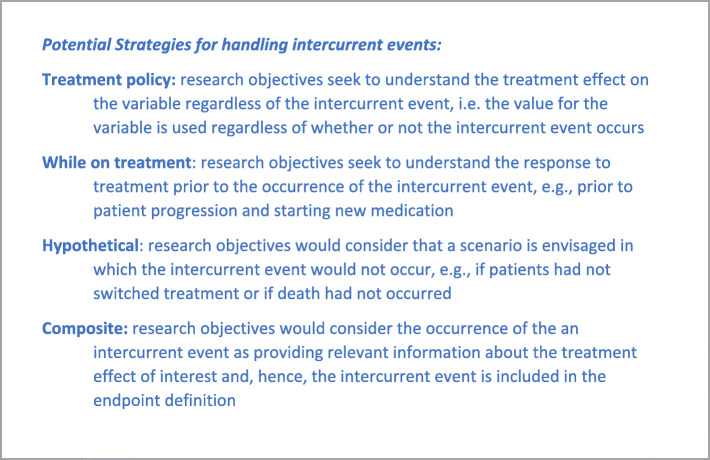
Definitions for strategies for handling intercurrent events from ICH E9 (R1) addendum [2]

The definition of an intercurrent event is a concept introduced in the estimand framework which may be new to many researchers. The addendum defines intercurrent event as events occurring after treatment initiation that affect either the interpretation or the existence of the measurements associated with the clinical question of interest. Such events arise from various patient journeys during the course of the study. For example, if we are interested in a PRO endpoint at month 6, some patients will still be on-treatment and their PRO result will reflect 6 months of treatment. Others may discontinue treatment due to an adverse event and start a new therapy – in this case, the assessment at month 6 reflects the effect of the initial treatment followed by a new therapy. Some patients may die prior to month 6 precluding the observation of the endpoint. Intercurrent events such as discontinuation due to adverse event, start of new therapy or death can all affect the interpretation of the PRO endpoint at month 6 or preclude its existence. As a result, each should be carefully considered when defining the treatment effect of interest. Common strategies to handle intercurrent events as proposed in the ICH E9 (R1) addendum are summarized in Fig. 2. Various strategies correspond to different questions of interest; for example, applying a treatment policy strategy for the start of new of therapy, the researcher is interested in assessing the effect on the PRO regardless of whether a patient is still on study treatment or receives new medication outside of the trial. Alternatively, the researcher may be interested in the effect on the PRO prior to the start of new therapy (while on treatment strategy) or only if a patient had not received new medication (hypothetical strategy). Further details with examples are provided in Section 3.

## Example of how to build an estimand for a PRO objective

**Fig. 3:**
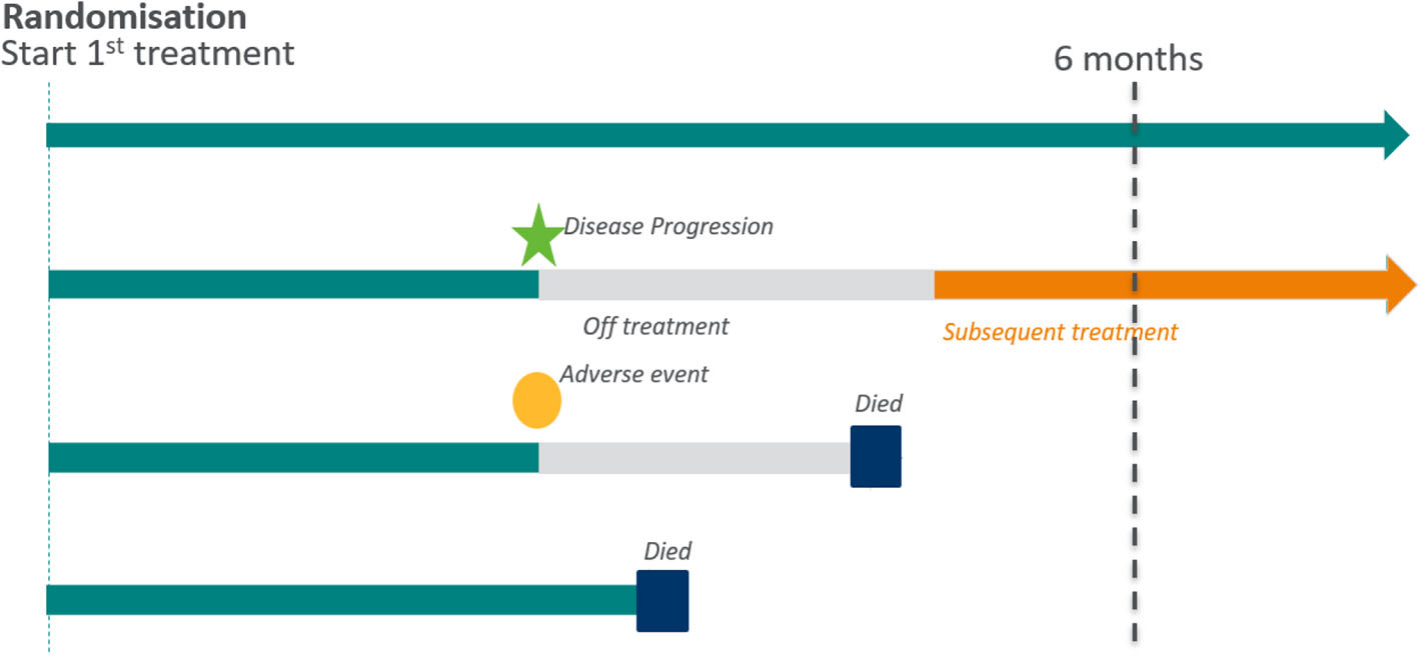
Illustrative Patient treatment journeys

Firstly, we want to consider the patients – who are the target population of interest and what do their treatment journeys look like? In designing clinical trials, we ideally want the scientific questions that we are asking to be directly relevant to real patient experiences. Figure 3 shows a schematic of four potential patient journeys in an oncology setting. After starting study treatment, patients generally receive treatment until disease progression. However, they may discontinue study treatment earlier, e.g. due to adverse events, and they may start a new anticancer therapy afterwards. The example below illustrates how considerations of various patient journeys impact the precise definition of the scientific question of interest and help to identify intercurrent events.

To illustrate the use of the estimand framework in creating a detailed clinical trial objective we shall begin with a naïve objective:

***“What is the effect of treatment X on patient’s quality of life?”***

It is important to clearly define a specific patient outcome of interest, i.e., to define the “variable of interest” []. Simply referring to a “PRO score” is not specific. In our naïve example, we have selected pain severity as the PRO variable of interest.

In addition, one should consider a relevant timeframe to evaluate outcomes when defining the PRO variable of interest. In this simple example, we have suggested a fixed timepoint (6 months post randomisation) at which to evaluate the variable.



Defining the parts of the estimand relating to the population of interest [] and treatment comparisons [] are presented next and thus to build this estimand:



The ICH E9 terminology uses the phrase “a summary measure” []. It is important to note that in this context the summary measure refers to the statistical summary approach, rather than just the measure or PRO instrument used. The summary measure should be defined; this could be the difference in the mean score between the two groups, or the proportion of patients in each group who have had meaningful improvement in symptoms. In this example, we suggest comparing the difference in mean scores between treatment groups.



Now we turn to intercurrent events, which are at the heart of the estimand framework and involve intricacies that can lead us to define different estimands. For the purposes of this illustrative example, we will consider two potential intercurrent events of interest commonly observed in oncology trials which are also important for PRO endpoints: discontinuation of treatment and patient deaths.

### Intercurrent event #1: treatment discontinuation

Different questions arise when considering treatment discontinuation prior to the planned assessment timeframe (e.g. 6 months post-randomization) that correspond to different potential strategies to handle this intercurrent event:

- Are we interested in the effect of treatment at 6 months post-randomization regardless of whether a patient is still on study treatment? If so, this would be illustrative of using a ***treatment policy strategy*** to handle the intercurrent event of treatment discontinuation. Note that this would require collection of the EORTC QLQ-C30 pain scores up to the 6-month period, even after treatment discontinuation (and disease progression and potential subsequent therapy) so that the value of the EORTC QLQ-C30 pain score at 6 months can be directly used in the analysis.
- Is it relevant to assess the effect only while receiving study treatment? If so, then this is illustrative of using a ***while on treatment strategy*** for this intercurrent event. Here, the EORTC QLQ-C30 pain score at 6 months is included in the analysis only if the patient is still on treatment at 6 months. Otherwise, the pain score assessed at the time of treatment discontinuation is used. Therefore, data collection after treatment discontinuation would not be required.
- Are we interested in the effect in the hypothetical scenario if all patients had received study medication for 6 months? If “yes”, then the approach to handling this would be considered the ***hypothetical strategy***. This can be appropriate strategy if the intercurrent event is not expected to occur in the target population in general. However, this strategy may not be a realistic assumption to be made with regards to PRO data in general.

### Intercurrent event #2: death

- Handling death events with a ***treatment policy*** strategy implies that the endpoint is considered regardless of deaths, which is simply impossible and as such would never be a plausible strategy for handling this type of intercurrent event for PRO data.
- Using a ***while on treatment*** **strategy** to handle deaths as an intercurrent event implies that we are interested in the effect on PROs until death. It is necessary to decide how to use these data, such as using the last observation prior to death if patients die prior to the time of interest for the endpoint (e.g., 6 months).
- A ***hypothetical strategy*** for handling an intercurrent event of death is when we envisage a hypothetical scenario where death would not have occurred and may be considered a reasonable approach for PRO endpoints if the number of patient deaths is expected to be low and that the patients deaths are not expected to be related to treatment (in the timescale of interest for the assessment of PRO endpoint). In this case we can use some assumptions to impute a reasonable value for the patient for the purpose of analysis.

For just these two intercurrent events, strategies for handling intercurrent events could lead to at least five different potentially quite plausible estimands (as treatment policy for death is excluded as implausible). Furthermore, it is conceivable to consider other appropriate strategies for handling these intercurrent events. Both a composite endpoint or a principal stratum approach could also be applicable for a PRO concept of interest [6, 7]. In fact, death is frequently considered as an event in some time-to-deterioration of quality of life analysis implying a composite strategy [8].

In this example we are considering pain, and, therefore, it is also important to consider the impact of pain medication use (if permissible per clinical trial protocol) as a potential intercurrent event. Taking pain medication would not typically be considered as an intercurrent event for most primary efficacy endpoints of an oncology trial as it’s not expected to impact tumor progression. Concomitant pain medication use becomes an important intercurrent event when considering the endpoint of pain as it may impact on the measurement of pain. We would need to define whether we are interested in the effect of our treatment on pain only when no pain medication is taken or regardless of whether patients take pain medication. Current standard practice typically leads to handling pain medications use with a treatment-policy strategy. In this case, we don’t measure just the effect of our treatment on pain, but we collect data that include the effects of treatment and pain medications (if used). As a result, the use of pain medications becomes part of our definition of the “treatment” attribute of the estimand.

Similarly, if patients discontinue treatment and start new anticancer therapy prior to the planned assessment timeline at month 6, this could also be included in the treatment effect planned to be measured. In these cases, it is not enough to define a simple “treatment X” as part of the estimand, rather there is the need to be more specific and define “treatment X followed by any potential subsequent antineoplastic therapy and/or concomitant pain medication (as needed)”.

To return to the example estimand, let us add in potential subsequent treatments and any concomitant treatments to the treatment attribute [], and then we can clarify our intercurrent event approaches []; let us assume *a treatment policy* approach to handle treatment discontinuation and *while on treatment* to handle death were used Therefore, our estimand becomes:



It is important to ensure that the final clinical trial objective reflects required specificity and details that explain whether it is intended for the PRO objective to demonstrate statistical superiority or non-inferiority of treatment X compared with treatment Y [9]. Additionally, if there is published or well-accepted minimal threshold for clinically meaningful between-group difference, it should also be stated (e.g. a between-group difference in mean EORTC-QLQ C30 pain sore > 7 points) [10].

## Case study – gallium trial

In the context of the estimand framework, the primary objectives relating to the GALLIUM trial have been presented by Rufibach (PharmStats 2019) [11], along with proposed sensitivity analysis. Publicly available data from the same trial was selected as a case study here in order to consider ways in which key HRQoL endpoints could retrospectively be described in the estimand framework.

GALLIUM was an open-label, randomised phase III clinical trial of obinutuzumab-based and rituximab-based chemo-immunotherapy in patients with previously untreated advanced indolent non-Hodgkin lymphoma (ClinicalTrials.gov ID NCT01332968). The primary variable of interest was investigator-assessed progression-free survival [12]. A secondary objective as stated in the GALLIUM protocol was “To assess patient-reported outcomes (PROs) in both arms.” [13]

One of the PRO endpoints of interest stated on ClinicalTrials.gov was the change from baseline in FACT-Lym lymphoma subscale score (FACT LymS). This subscales measures “additional concerns” relating to symptoms relevant to patients with lymphoma, and a change from baseline score > = 3 points for a patient is recognised as minimally important difference. In this trial there was a 6 months induction treatment period where patients received chemo-immunotherapy, followed by up to 2 years of immunotherapy maintenance in patients who responded to induction therapy (30 months total).

Taking the patient-reported lymphoma-related symptoms, as measured by the FACT LymS score as the key PRO measurement, along with information from the trial design, we can propose an estimand as follows (when considering treatment discontinuation and deaths as intercurrent events):

Estimand 1:



In order to propose this estimand we have had to consider carefully the patient-reported outcome of interest – in this example we have made clear that we are focusing on the lymphoma-related symptoms reported by the patient as measured by the FACT-LymS score. We have also clearly stated the timeframe we are interested in considering – in this case it is all FACT-LymS scores for the planned full duration of therapy (i.e., Up to 30 months post randomisation). In this estimand interest centres on the “While on Treatment” scores only.

An alternative estimand of interest could be considering FACT-G LymS score up to the planned 30 months treatment period, irrespective of whether patients continued to take active treatment or not (a treatment policy approach). From a practical point of view this means that FACT-G LymS score would need to be collected even after treatment discontinuation (and potentially disease progression, if this happens prior to 30 months after randomisation). The collection of PRO data regularly post treatment discontinuation is not currently a common approach in oncology clinical trials. There are other considerations highlighted by this proposed estimand: what is the summary measure of interest – in this case the estimand describes the proportion of patients with an improvement – but is the timescale chosen of 30 months appropriate for this summary measure? An alternative could a summary measure relating to a mean change from baseline in score over time (up until maximum of 30 months) in each treatment group, as shown in Estimand 2.

Estimand 2:



## Conclusions

The estimand framework provides guidance on the key attributes for defining study objectives, including PRO objectives, that should be thoughtfully considered when designing a phase III clinical trial. A clear and specific PRO objective should be defined, enabling a precise estimand statement. Attributes of the estimand described in the framework help to ensure that a detailed PRO objective can be constructed. Even with one “naïve” objective, the use of the estimand framework provides a way to clearly discuss several very feasible estimands of interest, as illustrated above. One estimand could be chosen as the key approach; other ways of handling an intercurrent event could lead to different estimands and additional supplementary analyses. Using this staged approach to specify estimands should then enable clearer specification of PRO objectives in protocols and analysis plans of what sensitivity analysis should be considered, together with the data that needs to be collected to enable this.

These discussions with a case-study example, aim to highlight how the use of the attributes of the estimand provide potential ways to consider the detailed PRO objective of interest and build the related estimand. It also highlights with just one case-study example how different strategies can change how the estimand is written and how to more clearly interpret study results.

It is recognised that different stakeholders (e.g., patients, regulators, HTA authorities, and payers) have different objectives to answer from clinical trials conducted and that this may influence how trials should be designed and analysed. As examples, the long-term impact of adverse events on patient symptoms or PROs after radiological disease progression, or patient outcomes collected post-end of treatment, would each require consideration of different specific estimands and clear specification about what was needed to be measured in order to evaluate each PRO endpoint.

Once an estimand has been specified, we then need to move on to focus on the estimator which is the statistical technique with which you will derive your ‘estimate’ of the treatment effect. A well-defined estimand allows study teams to work constructively with statisticians to take a well-thought-out objective and to choose an appropriate analysis that need to be applied. However, the specifics and appropriateness of these estimators and a thorough discussion of their selection is out of the scope of this work.

Experience with the estimand framework is growing in clinical research across various therapeutic areas [11, 14–18], as well as its value as a providing a common language to facilitate discussions with all relevant stakeholders in clinical trials. In the FDA’s Patient Focused Drug Development (PFDD) workstream, the PFDD draft Guidance 4 focuses on “Incorporating Clinical Outcome Assessments into Endpoints for Regulatory Decision Making” [19], and includes discussion of the estimand framework and a case study example considering the PRO of physical function in breast cancer.

However, the estimand framework is not just a new language for defining trial objectives – it will change the way in which clinical trials are designed, analyzed, and interpreted, as well as how the data collection strategy is informed. By developing precise objectives relating to assessing the patient’s perspective of treatment and embracing the estimand framework, we can use the estimand framework to ensure useful collection of data in clinical trial protocols and provide clear interpretation and conclusions to all stakeholders, thereby lending to clear evaluation on the impact of treatment on patients’ lives.

## Data Availability

Not applicable.

## Abbreviations

FACT LymS: FACT-Lym lymphoma subscale score
HRQoL: Health Related Quality of Life
ICH: International Committee for Harmonization of Technical Requirements for Pharmaceuticals for Human Use
PFDD: Patient Focused Drug Development
PRO: Patient-Reported Outcome

## References

[1] Institute of Medicine (2001). Crossing the quality chasm: a new health system for the 21st Century.

[2] International Council for Harmonisation (2019). Addendum on estimands and sensitivity analysis in clinical trials to the guideline om statistical principles for clinical trials E9(R).

[3] Huang, X., Lin, J., & Demner-Fushman, D. (2006). Evaluation of PICO as a knowledge representation for clinical questions. *AMIA Annual Symposium Proceedings, 2006*, 359–363. PMC1839740 17238363

[4] Bottomley A, Pe M, Sloan J (2016). Analysing data from patient-reported outcome and quality of life endpoints for cancer clinical trials: a start in setting international standards. The Lancet Oncology.

[5] Coens C, Pe M, Dueck AC (2020). International standards for the analysis of quality of life and patient reported outcomes endpoints in cancer randomised controlled trials; recommendations based on critical reviews of the literature and international multi-expert, multi-stakeholder collaborative process. The Lancet Oncology.

[6] Uemura Y, Taguri M, Kawahara T, Chiba Y (2019). Simple methods for the estimation and sensitivity analysis of principal strata effects using marginal structural models: Application to a bone fracture prevention trial. Biometrical Journal.

[7] Rubin DB (2006). Causal inference through potential outcomes and principal stratification: Application to studies with “censoring” due to death. Statistical Science.

[8] Anota A, Hamidou Z, Paget-Bailly S (2015). Time to health-related quality of life score deterioration as a modality of longitudinal analysis for health-related quality of life studies in oncology: Do we need RECIST for quality of life to achieve standardization?. Quality of Life Research.

[9] Bell J. Implementing estimands in trials: detailed clinical objectives. Presented at PSI Conference June 2019.

[10] Cocks K, King MT, Velikova G (2012). Evidence-based guidelines for interpreting change scores for the European organisation for the research and treatment of Cancer quality of life questionnaire Core 30. European Journal of Cancer.

[11] Rufibach K (2019). Treatment effect quantification for time-to-event endpoints–Estimands, analysis strategies, and beyond. Pharmaceutical Statistics.

[12] Marcus R, Davies A, Ando K (2017). Obinutuzumab for the first-line treatment of follicular lymphoma. The New England Journal of Medicine.

[13] Davies, A., Trask, P., Demeter, J., et al. (2020). Health-related quality of life in the phase III GALLIUM study of obinutuzumab-or rituximab-based chemotherapy in patients with previously untreated advanced follicular lymphoma. *Annals of Hematology*, 1–10. 10.1007/s00277-020-04021-6 PMC7683459 32314038

[14] Aroda VR, Saugstrup T, Buse JB, Donsmark M, Zacho J, Davies MJ (2019). Incorporating and interpreting regulatory guidance on estimands in diabetes clinical trials: The PIONEER 1 randomized clinical trial as an example. Diabetes, Obesity & Metabolism.

[15] Degtyarev E, Zhang Y, Sen K (2019). Estimands and the patient journey: Addressing the right question in oncology clinical trials. JCO Precision Oncology.

[16] Callegari F, Akacha M, Quarg P, Pandhi S, von Raison F, Zuber E (2020). Estimands in a chronic pain trial: Challenges and opportunities. Statistics in Biopharmaceutical Research.

[17] Ratitch B, Goel N, Mallinckrodt C (2020). Defining efficacy estimands in clinical trials: Examples illustrating ICH E9 (R1) guidelines. Therapeutic Innovation & Regulatory Science.

[18] Ratitch B, Bell J, Mallinckrodt C (2020). Choosing estimands in clinical trials: Putting the ICH E9 (R1) into practice. Therapeutic Innovation & Regulatory Science.

[19] Patient-focused drug development guidance public workshop: Incorporating clinical outcome assesssments into endpoints for regulatory decision making (https://www.fda.gov/drugs/development-approval-process-drugs/public-workshop-patient-focused-drug-development-guidance-4-incorporating-clinical-outcome). Published 2019. Accessed Dec 2019.

